# Diagnostic potential of urinary CX3CL1 for amnestic mild cognitive impairment and Alzheimer’s disease

**DOI:** 10.3389/fnagi.2025.1501762

**Published:** 2025-01-23

**Authors:** Yali Xu, Jie Zhang, Ying-Ying Shen, Wei-Wei Li, Bin Li, Hai-Ping Cheng, Gui-Hua Zeng

**Affiliations:** ^1^Department of Geriatrics, Chongqing General Hospital, Chongqing University, Chongqing, China; ^2^Chongqing Clinical Research Centre for Geriatric Diseases, Chongqing, China; ^3^Department of Neurology and Center for Clinical Neuroscience, Daping Hospital, Army Medical University, Chongqing, China; ^4^Department of Health Management, Chongqing General Hospital, Chongqing University, Chongqing, China

**Keywords:** Alzheimer’s disease, aging, amnestic mild cognitive impairment, biomarker, cognitively normal subjects, CX3CL1, diagnosis, urinary

## Abstract

**Introduction:**

The role of the chemokine CX3CL1 in the processes of aging and Alzheimer’s disease (AD) pathogenesis is well-established. This study aims to evaluate the diagnostic potential of urinary CX3CL1 levels in distinguishing between AD patients, those experiencing amnestic mild cognitive impairment (aMCI), and cognitively normal (CN) individuals.

**Methods:**

A cohort comprising 516 CN individuals across various age groups, 102 AD patients, and 65 subjects with aMCI was assembled, alongside 93 age- and sex-matched CN controls. Enzyme-linked immunosorbent assay (ELISA) was utilized to quantify urinary CX3CL1 levels.

**Results:**

Urinary CX3CL1 concentrations exhibited an age-dependent increase and demonstrated a positive correlation with age. Comparatively, AD patients exhibited significantly elevated urinary CX3CL1 levels when contrasted with both the CN controls and the aMCI cohort. Conversely, aMCI patients displayed urinary CX3CL1 levels that were notably reduced in comparison to both the AD and CN groups.

**Conclusion:**

Urinary CX3CL1 levels correlate with the aging process and may serve as a potential diagnostic biomarker for both amnestic mild cognitive impairment (aMCI) and Alzheimer’s disease (AD).

## 1 Introduction

With the aging of the population, the incidence of cognitive disorders is increasing, among which amnestic mild cognitive impairment (aMCI) is considered as a pre-stage of Alzheimer’s Disease (AD), and the early identification and intervention of these two diseases are crucial. In recent years, there has been a growing interest in identifying robust biomarkers to facilitate the effective tracking and treatment of cognitive decline.

Inflammation plays an integral role in AD, and chemokine CX3CL1 (C-X3-C motif ligand 1, aka Fractalkine), emerging as a key molecule linking inflammatory and neuroprotective mechanisms within the central nervous system, play a special role in the development of AD (Zhang et al., 2021; Bivona et al., 2023; Pawelec et al., 2020) and aging (Pawelec et al., 2020; Mecca et al., 2018).

Previous studies have focused on changes in CX3CL1 levels in biological samples such as blood or cerebrospinal fluid (CSF) (Kulczyńska-Przybik et al., 2020; Zhou et al., 2023; Nordengen et al., 2023). However, the value of urine, as a relatively easy-to-obtain and non-invasive biological sample, in reflecting dynamic changes in CX3CL1 *in vivo* remains to be comprehensively investigated. In present study, we aimed to detect the urinary CX3CL1 levels in patients with aMCI, patients with AD and normal cognitive function subjects, specifically explored the variability of urinary CX3CL1 levels among individuals of different ages.

## 2 Method

The research was approved by the Medical Ethics Committee of Chongqing General Hospital. Informed consent was obtained from the participants or their respective legal representatives.

### 2.1 Study population

A total of 776 participants, including 516 cognitively normal (CN) participants across a spectrum of ages, as well as a cohort of 102 individuals with AD, 65 with aMCl and 93 age- and sex-matched CN controls, all were recruited from Chongqing General Hospital and Daping Hospital. All urinary samples were collected between 2014 and 2024 from the two hospitals (Chongqing General Hospital and Daping Hospital in Chongqing, China).

The exclusionary criteria were defined by the presence of: (i) a familial dementia history; (ii) chronic psychiatric conditions, including bipolar disorder, schizophrenia, or substance-related disorders; (iii) a past traumatic brain injury or additional neurological comorbidities; (iv) critical medical illnesses, such as advanced pulmonary, cardiac, hepatic, or renal dysfunctions, or malignancies; and (v) current urinary tract infection.

### 2.2 Clinical assessment

The procedural was reported in our previous study (Xu et al., 2020). All participants underwent a comprehensive clinical assessment encompassing medical history review, physical examination, neuropsychological evaluations, and laboratory analyses. Cognitive and functional capabilities were gauged using a suite of neuropsychological instruments, including the Chinese Mini-Mental State Examination (MMSE), Montreal Cognitive Assessment, and Activities of Daily Living (ADL) assessments. Those who exhibited abnormal findings on the MMSE or Montreal Cognitive Assessment were further evaluated using an extended neuropsychological test battery, comprising the Auditory Verbal Learning Test, Clock Drawing Test, Trail Making Test, Boston Naming Test, Digit Span Test, Clinical Dementia Rating (CDR), Pfeiffer Outpatient Disability Questionnaire, and Hachinski Ischemic Score. Subjects presenting with cognitive impairment underwent additional diagnostic procedures, including brain CT/MRI scans and blood tests to measure levels of thyroxine, vitamin B12, folic acid, and to screen for human immunodeficiency virus/syphilis infections, aiming to exclude metabolic or infectious causes of cognitive deterioration. The diagnosis of amnestic mild cognitive impairment (aMCI) was established following the Petersen criteria (Petersen, 2004). Dementia was diagnosed using criteria adapted from the Diagnostic and Statistical Manual of Mental Disorders, Fourth Edition, while AD was diagnosed in accordance with the guidelines set by the National Institute of Neurological and Communicative Diseases and Stroke/AD and Related Disorders Association (McKhann et al., 1984).

### 2.3 Sample collection

Post-collection, urine specimens were centrifuged within a 2-h. Subsequently, the resulting aliquots were promptly cryopreserved at −80°C for subsequent analytical procedures.

### 2.4 Urinary CX3CL1 measurement

The concentration of CX3CL1 was measured using enzyme-linked immunosorbent assay kits (R&D Systems, Minneapolis, MN, USA). To account for variations in water intake, renal function, and urinary retention that might affect CX3CL1 levels, urinary CX3CL1 concentrations were adjusted relative to creatinine levels. The adjusted levels were reported as nanograms per milligram of creatinine.

### 2.5 Statistical analysis

Data normality was evaluated using the Kolmogorov–Smirnov test. For continuous variables across multiple groups, one-way ANOVA was applied for normally distributed data, while the Kruskal–Wallis test was utilized for non-normally distributed data. Categorical data comparisons were conducted via Fisher’s exact test or chi-squared test. Correlations between CX3CL1 levels and cognitive scores or age were determined using Pearson or Spearman correlation analyses. The optimal sensitivity and specificity were determined using receiver operating characteristic curve analysis with a non-parametric approach. Data are presented as mean ± standard deviation (SD). Statistical significance was set at *p* < 0.05, and analyses were conducted using SPSS version 25.0.

### 2.6 Data availability

Anonymized data will be shared on request from a qualified investigator.

## 3 Results

### 3.1 CX3CL1 concentrations in the urine of CN individuals across various age groups

**Figure 1:**
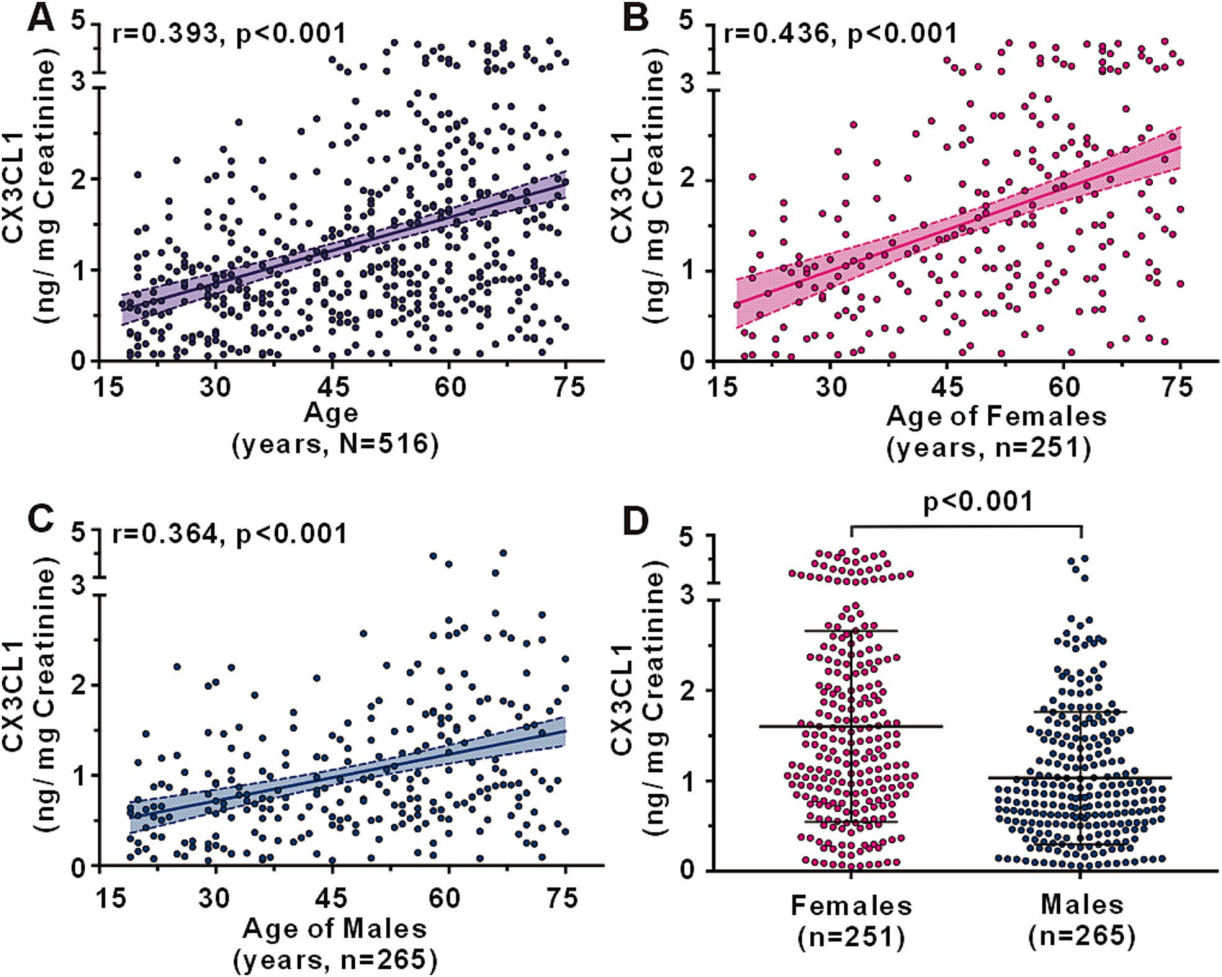
Urinary CX3CL1 levels across ages in cognitively normal subjects. Correlation between urinary CX3CL1 levels and age in all subjects **(A)**, females **(B)**, and males **(C)**. Comparison of urinary CX3CL1 levels between females and males **(D)**.

A total of 516 CN individuals across a spectrum of ages were recruited to examine the alterations in urinary CX3CL1 levels associated with aging (Supplementary Table S1). No significant age differences were observed between the female (*n* = 251) and male (*n* = 265) groups, with average ages of 49.74 ± 15.56 and 48.17 ± 16.08 years, respectively (*p* = 0.306). Urinary CX3CL1 levels were found to increase with age, exhibiting a positive correlation in the entire cohort (*r* = 0.393, *p* < 0.001) (Figure 1A), as well as in females (*r* = 0.436, *p* < 0.001) (Figure 1B) and males (*r* = 0.364, *p* < 0.001) (Figure 1C). In different age groups, young age groups (Group age 18–29 and Group age 30–39) have lower urinary CX3CL1 levels than in older age groups (Group age 60–69 and Group age 70–75) in all subjects (Supplementary Figure S1A), females (Supplementary Figure S1C), males (Supplementary Figure S1D). Urinary CX3CL1 levels were significantly elevated in females compared to males (1.602 ± 1.057 vs.1.031 ± 0.735 ng/mg creatinine, *p* < 0.001) (Figure 1D). In middle age group and elder age group, subjects 40 years of age and older, urinary CX3CL1 values were greater in females than in males (Supplementary Figure S1B). Nevertheless, no significant differences in urinary CX3CL1 levels were observed between female and male subjects younger than 40 years of age (Supplementary Figure S1B).

### 3.2 Urinary CX3CL1 levels in patients with AD and aMCI, and CN subjects

A cohort comprising AD patients (*n* = 102), aMCI patients (*n* = 65), and age- and sex-matched cognitively normal (CN) controls (*n* = 93) was assembled to assess urinary CX3CL1 level variations across the groups (Table 1). No significant differences were noted in terms of age, sex, education level, urinary creatinine levels, or prevalence of comorbidities such as diabetes mellitus, hypertension, and hypercholesterolemia among the AD, aMCI, and CN groups. AD patients exhibited the lowest Mini-Mental State Examination (MMSE) scores and the highest Clinical Dementia Rating (CDR) and Activities of Daily Living (ADL) scores compared to the other two groups. aMCI patients demonstrated lower MMSE scores and higher CDR and ADL scores when compared to CN subjects.

**Table 1:** Demographic and clinical data of the AD, aMCI and CN subjects.

Clinical variables	AD (*n* = 102)	aMCI (*n* = 65)	CN (*n* = 93)	*p*-value
Age (years), mean ± SD	72.64 ± 8.67	71.03 ± 9.45	70.61 ± 8.15	0.4327
≤75 years (%)	65 (63.73)	42 (64.62)	64 (68.82)	0.7366
>75 years (%)	37 (36.27)	23 (35.38)	29 (31.18)	0.7366
Education (years)	7.186 ± 3.350	8.68 ± 3.231	9.548 ± 3.239	<0.001
Female, *n* (%)	50 (49.02)	32 (49.23)	50 (53.76)	0.7710
MMSE score, mean ± SD	14.17 ± 6.605	22.68 ± 2.431	27.75 ± 1.434	<0.001
CDR score, mean ± SD	1.892 ± 0.866	0.50	0	<0.001
ADL score, mean ± SD	49.34 ± 15.36	34.14 ± 11.70	21.38 ± 1.687	<0.001
Hyperlipidaemia, *n* (%)	34 (33.33)	16 (24.62)	20 (21.51)	0.1577
Hypertension, *n* (%)	50 (49.02)	29 (44.62)	35 (37.63)	0.2750
Diabetes mellitus, *n* (%)	24 (23.53)	9 (13.85)	11 (11.83)	0.0699
Creatinine (mg/L), Mean ± SD	971.90 ± 591.3	977.4 ± 669.6	938.3 ± 635.3	0.8953

AD, Alzheimer’s disease; ADL, Activities of Daily Living; aMCI, amnestic mild cognitive impairment; CDR, Clinical Dementia Rating; CN, cognitively normal controls; MMSE, Mini-Mental State Examination; SD, standard deviation.

**Figure 2:**
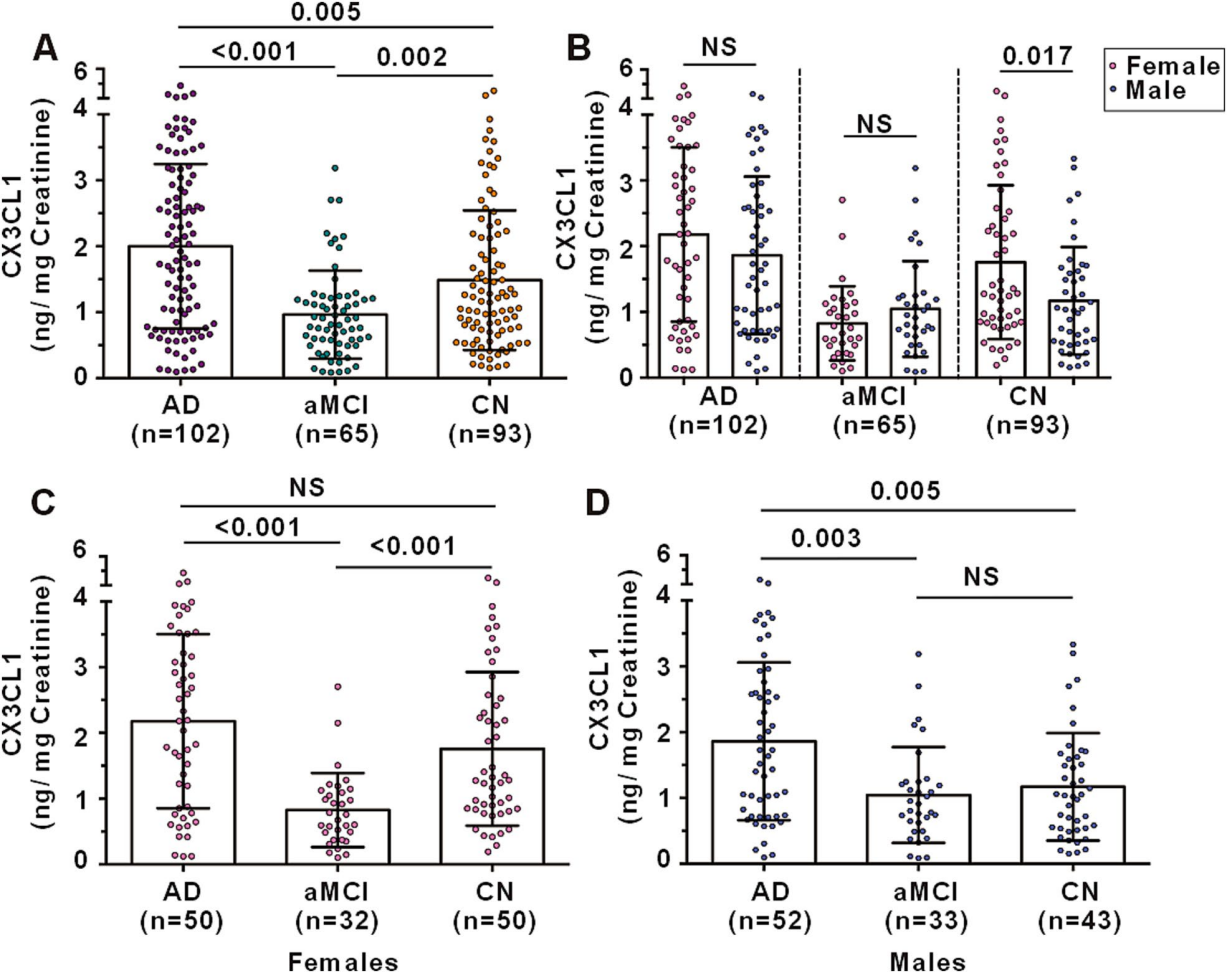
Urinary CX3CL1 levels among AD, aMCI and CN subjects. **(A)** Comparison of urinary CX3CL1 levels among AD, aMCI and CN subjects. **(B)** Comparison of urinary CX3CL1 levels between females and males in AD, aMCI and CN groups. **(C)** Comparison of urinary CX3CL1 levels in females among AD, aMCI and CN groups. **(D)** Comparison of urinary CX3CL1 levels in males among AD, aMCI and CN groups. NS denotes non-statistically significant.

Urinary CX3CL1 levels varied significantly among the groups. AD patients had elevated urinary CX3CL1 levels compared to CN controls (1.999 ± 1.248 vs. 1.486 ± 1.058 ng/mg creatinine, *p* = 0.005) and aMCI patients (1.999 ± 1.248 vs. 0.965 ± 0.667 ng/mg creatinine, *p* < 0.001). Conversely, aMCI patients showed reduced urinary CX3CL1 levels relative to CN controls (0.965 ± 0.667 vs. 1.486 ± 1.058 ng/mg creatinine, *p* = 0.002) (Figure 2A).

In the CN group, females exhibited higher urinary CX3CL1 levels compared to males (1.757 ± 1.169 vs. 1.170 ± 0.8167 ng/mg creatinine, *p* = 0.017) (Figure 2B). However, no significant differences in urinary CX3CL1 levels were observed between females and males in the AD and aMCI groups (Figure 2B). Among females, AD patients and CN subjects had higher urinary CX3CL1 levels than aMCI patients, with no significant difference between AD patients and CN subjects (Figure 2C). Among males, AD patients had higher urinary CX3CL1 levels than both aMCI and CN subjects, with no significant difference between aMCI and CN subjects (Figure 2D).

### 3.3 Associations between urinary CX3CL1 levels and cognitive assessment scores

**Figure 3:**
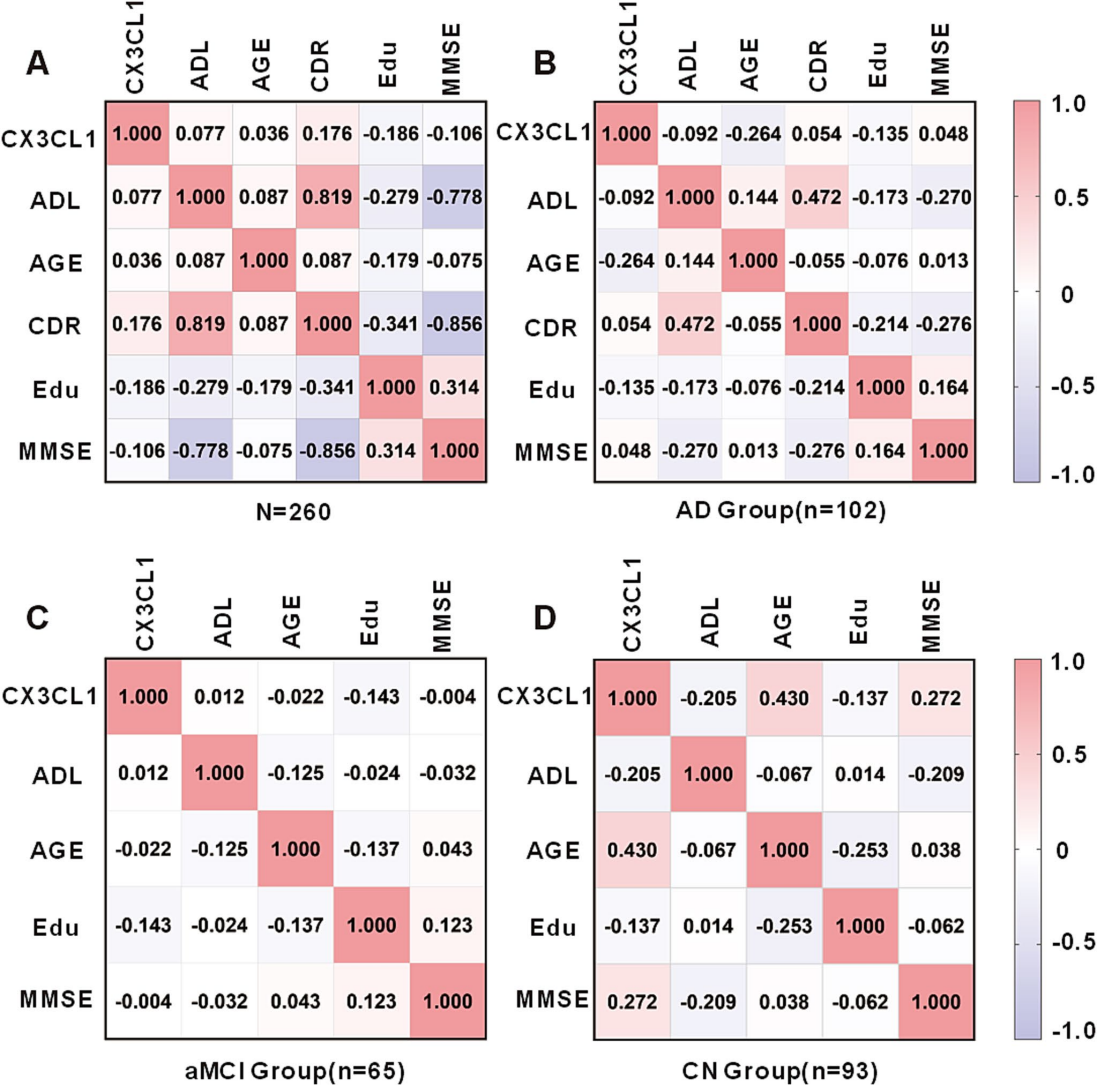
Correlation matrix showing Spearman’s correlations between urinary CX3CL1 levels, ADL scores, Age, CDR scores, Education and MMSE scores in the cohort including the AD, aMCI and CN groups **(A)**, AD group **(B)**, aMCI group **(C)**, and CN group **(D)**.

**Figure 4:**
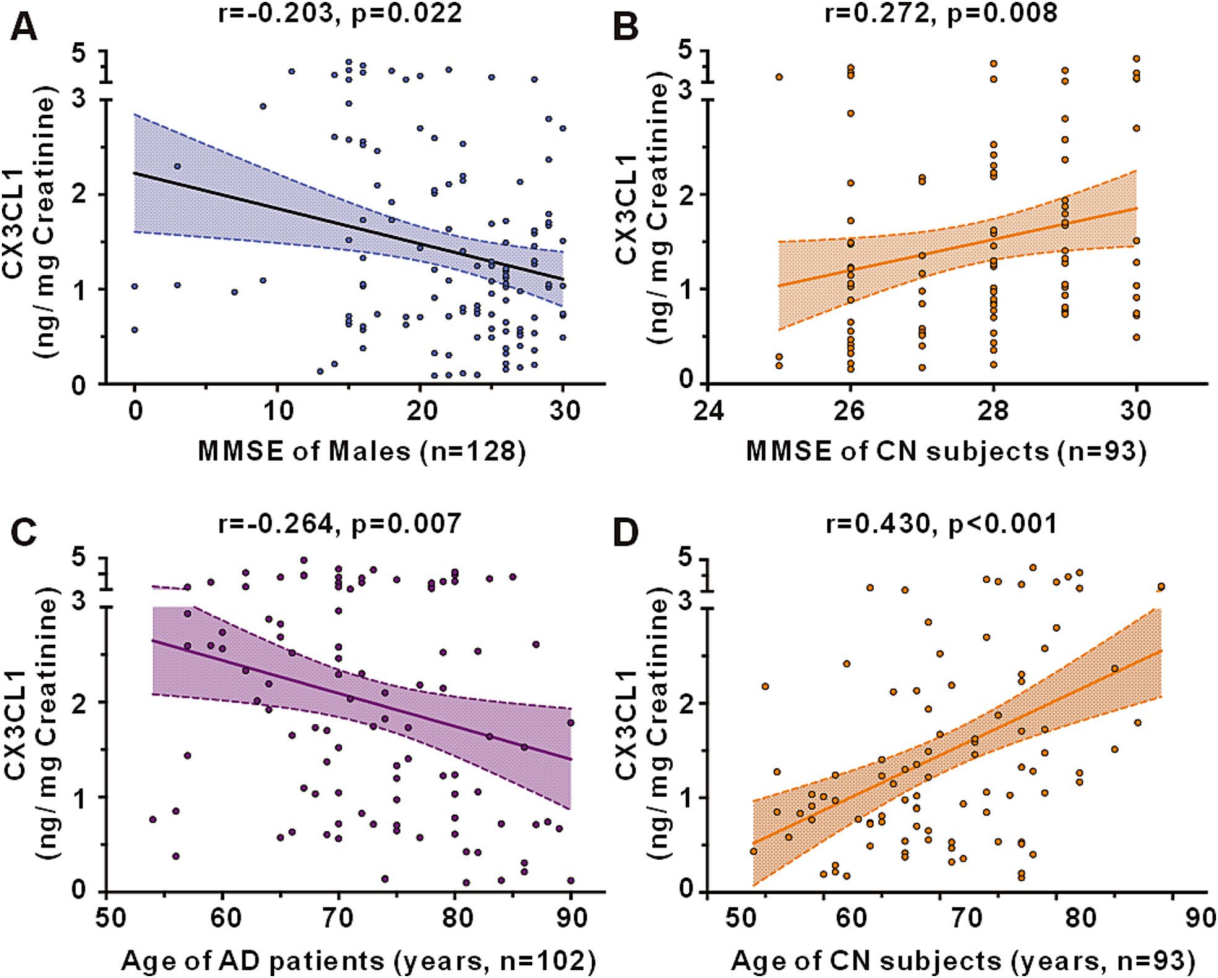
Correlations between urinary CX3CL1 levels and MMSE scores or age in the cohort including the AD, aMCI and CN groups. **(A)** Correlation between urinary CX3CL1 levels and MMSE scores in males of the cohort including the AD, aMCI and CN groups. **(B)** Correlation between urinary CX3CL1 levels and MMSE scores in CN groups. **(C)** Correlation between urinary CX3CL1 levels and age in AD group. **(D)** Correlation between urinary CX3CL1 levels and age in CN group.

In a cohort encompassing AD patients, aMCI patients, and CN subjects, urinary CX3CL1 levels showed no correlation with MMSE scores among all participants (Figure 3A) and in females (Supplementary Figure S2A females). However, a significant negative correlation was observed between urinary CX3CL1 levels and MMSE scores in males (*r* = −0.203, *p* = 0.022) (Figure 4A and Supplementary Figure S2A).

Urinary CX3CL1 concentrations did not correlate with MMSE scores in patients with AD (Figure 3B), the AD female subgroup, and the AD male subgroup (Supplementary Figure S2B). Urinary CX3CL1 levels did not correlated with MMSE scores in patients with aMCI (Figure 3C), the aMCI female subgroup and the aMCI male subgroup (Supplementary Figure S2C).

Urinary CX3CL1 levels were positively correlated with MMSE scores in the CN group (*r* = 0.272, *p* = 0.008) (Figures 3D, 4B). Furthermore, urinary CX3CL1 levels did not correlated with MMSE scores in the CN female subgroup and the CN male subgroup (Supplementary Figure S2D).

### 3.4 Association between urinary CX3CL1 levels and age by group

Within a cohort that included AD patients, aMCI patients, and CN subjects, no correlation was found between urinary CX3CL1 levels and age across all participants (Figure 3A), in females and in males (Supplementary Figure S2A). Urinary CX3CL1 levels were negatively correlated with age in AD patients (*r* = −0.264, *p* = 0.007) (Figures 3B, 4C) and in the male AD subgroup (*r* = −0.299, *p* = 0.031) (Supplementary Figure S2B), while no such correlation was observed in the female AD subgroup (Supplementary Figure S2B). Urinary CX3CL1 levels did not correlated with age in aMCI patients (Figure 3C), the male aMCI subgroup and the female aMCI subgroup (Supplementary Figure S2C). Urinary CX3CL1 levels were positively correlated with age in CN group (*r* = −0.430, *p* < 0.001) (Figures 3D, 4D), in the female CN subgroup (*r* = 0.523, *p* < 0.001) (Supplementary Figure S2D), and in the male CN subgroup (*r* = 0.464, *p* = 0.002) (Supplementary Figure S2D).

### 3.5 Diagnostic potential of CX3CL1 for AD and aMCI

**Figure 5:**
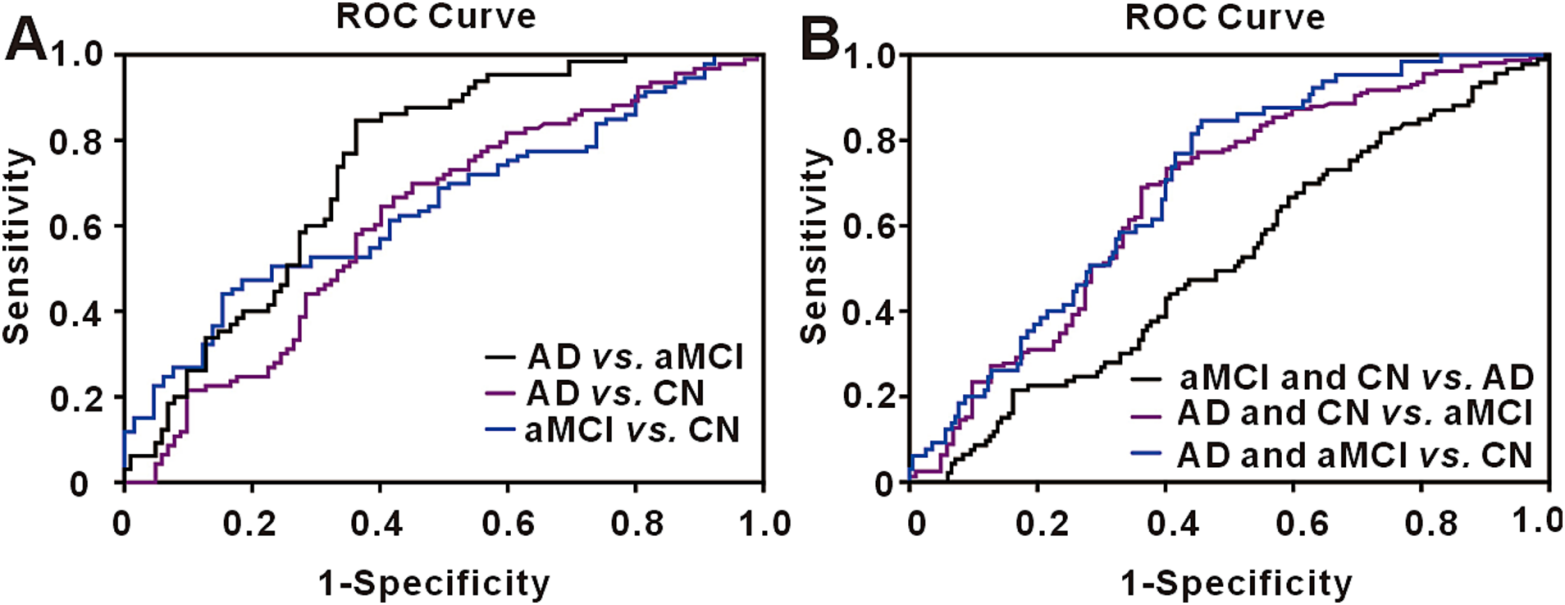
Receiver operating characteristic (ROC) curve for urinary CX3CL1. **(A)** The ROC curve for urinary CX3CL1 in discriminating AD from aMCI, AD from CN and aMCI from CN. **(B)** The ROC curve for urinary CX3CL1 in discriminating aMCI and CN from AD, AD and CN from aMCI, AD and aMCI from CN.

The area under the receiver operating characteristic curve (AUC) for urinary CX3CL1 in distinguishing AD from CN was calculated at 0.6174 (95% confidence intervals [CI], 0.5384–0.6964). With the cutoff value optimized via Youden’s index, urinary CX3CL1 achieved 69.89% sensitivity and 54.90% specificity in identifying AD versus CN (refer to Figure 5A and Supplementary Table S2 for details). The diagnostic accuracy of urinary CX3CL1 for distinguishing AD from aMCI was indicated by an AUC of 0.7412, with sensitivities and specificities reaching 84.62 and 63.73%, respectively (Figure 5A and Supplementary Table S2). In contrast, the AUC for differentiating aMCI from CN subjects was 0.6452, with sensitivities and specificities of 47.31 and 81.54%, respectively (Figure 5A and Supplementary Table S2).

We conducted additional analyses to assess the capacity of urinary CX3CL1 to distinguish aMCI from both AD and CN subjects (Figure 5B and Supplementary Table S2). The AUC for this comparison was 0.6954 (95% CI, 0.6289–0.7618), with sensitivities and specificities of 84.62 and 54.36%, respectively. Similarly, we evaluated the potential of urinary CX3CL1 to differentiate AD from the combined aMCI and CN groups (Figure 5B and Supplementary Table S2). The resultant AUC was 0.6683 (95% CI, 0.5985–0.7381), accompanied by sensitivities and specificities of 73.42 and 59.80%, respectively.

## 4 Discussion

To the best of our knowledge, this study represents the first to explore the association between urinary CX3CL1 levels and the process of aging, as well as to compare these levels between aMCI patients and AD patients. Our findings indicate a positive association between urinary CX3CL1 levels and age in cognitively normal individuals. Notably, AD patients demonstrated markedly higher urinary CX3CL1 levels compared to both aMCI patients and CN subjects, while aMCI patients showed significantly lower levels than those observed in the AD and CN groups.

CX3CL1 is recognized for its significant role in the aging process (Eugenin et al., 2023), age has a strong influence on blood cytokine CX3CL1 concentration (Compte et al., 2024). In our study, urinary CX3CL1 levels increased with aging, urinary CX3CL1 levels also increased with aging in normally cognitive function elderly subjects, which is an important risk factor for AD (Hou et al., 2019). Females exhibited higher urinary CX3CL1 concentrations compared to males, and indeed CX3CL1 is different between females and males (Pappritz et al., 2023).

CX3CL1 is an independently associated biomarker of AD (Trares et al., 2022). Some previous studies, mostly on blood and CSF CX3CL1 in AD or MCI. Most studies found that elevated CX3CL1 levels in both CSF and blood among individuals with AD and aMCI, surpassing those found in cognitively normal subjects. These levels have been correlated with the severity of the disease and the cognitive deterioration observed in AD patients (Kulczyńska-Przybik et al., 2020; Trares et al., 2022; Bivona et al., 2022; Kulczyńska-Przybik et al., 2023). However, a few analyses have reported opposite findings (Perea et al., 2018). A meta-analysis shows that the blood CX3CL1 levels are not significant different between AD patients and control subjects, yet the large effect size in MCI compared to controls suggests its potential as a biomarker to distinguish MCI patients from healthy individuals (Zhou et al., 2023). Blood CX3CL1 levels are reportedly higher level in MCI than in controls, with MCI patients exhibiting over twice the CX3CL1 levels compared to controls and a 13% lower level of CX3CL1 in AD compared to MCI (Zhou et al., 2023).

In this study, we firstly showed that patients with aMCI exhibit reduced urinary CX3CL1 levels compared to CN subjects, whereas AD patients display elevated levels relative to CN subjects. Additionally, a positive association was observed between urinary CX3CL1 levels and MMSE scores in the cognitively normal elderly subjects. While urinary CX3CL1 levels decreased with cognitive decline, aMCI had lower urinary CX3CL1 levels than cognitively normal elderly subjects. Along with the progression of dementia, urinary CX3CL1 levels increased with the progression of AD.

Our results highlight a variable pattern in urinary CX3CL1 levels across the cognitive impairment spectrum, with a notable decrease in aMCI patients, followed by an increase in those with AD. The ROC curve analysis revealed a larger area for CX3CL1 in aMCI subjects compared to traditional AD markers, suggesting its potential in early identification of aMCI and AD. These findings suggest that urinary CX3CL1 could serve as a biomarker to distinguish between cognitively normal elderly and those with aMCI, as well as to track disease progression.

It is relatively straightforward to comprehend the changes in urinary CX3CL1 when there are alterations in cognitive function. CX3CL1 is expressed on neuronal surfaces as a membrane-bound protein (mCX3CL1), which can be processed by extracellular proteolysis into multiple soluble CX3CL1 (sCX3CL1) isoforms. These distinct CX3CL1 forms can either exert anti-inflammatory effects on microglia in some contexts or promote inflammation in others, exacerbating neurologic conditions. Shifts in the equilibrium of CX3CL1 forms may represent a mechanism that links aging and AD pathogenesis (Eugenin et al., 2023). CX3CL1, interacting with its receptor CX3CR1, is pivotal in AD pathogenesis (Finneran and Nash, 2019). This chemokine, expressed by neurons, exists as a membrane-bound form (mCX3CL1) and can be cleaved to generate soluble forms (sCX3CL1), which can have either anti-inflammatory or pro-inflammatory effects on microglia, thereby influencing neurological disorders (Finneran and Nash, 2019).

The field of neurological disease biomarker research has identified urine as an important source of biomarkers. Urine is an easily accessible biomarker source (Benatar et al., 2016). Urine is convenience and non-invasive to collect. Urinary biomarkers offer a less invasive and more straightforward alternative to CSF for diagnostic measures, with urine being easier to collect and less complex for analysis compared to blood or CSF (Shepheard et al., 2017; Jia et al., 2017). Urinary CX3CL1’s potential as an early. Urinary CX3CL1’s potential as an early biomarker holds diagnostic and prognostic significance, reflecting changes across the AD spectrum, from preclinical to prodromal and dementia stages.

A key merit of our investigation was its pioneering approach in examining the diagnostic potential of urinary CX3CL1 across two southwestern Chinese centers. However, the study is not without limitations. Notably, AD and aMCI diagnoses lacked pathological validation, such as through amyloid Positron Emission Tomography (PET) scans or CSF biomarkers. Our and others’ research suggests that a significant proportion of patients diagnosed with probable AD based solely on clinical signs may not exhibit brain amyloid plaques, thus questioning their AD status (Li et al., 2019; Ossenkoppele et al., 2015). Additionally, we did not assess CX3CL1 levels in blood or CSF, which precludes us from evaluating the comparative diagnostic efficacy of urinary CX3CL1 against these sources or exploring its correlation with established biomarkers like Amyloid-*β* (Aβ) and phosphorylated tau (p-tau). Lastly, the cross-sectional nature of our study limits the inference of causality; hence, longitudinal studies are therefore required to address this issue.

In conclusion, our investigation establishes that urinary CX3CL1 can effectively distinguish between aMCI and AD patients and cognitively normal individuals. This dedicates that urinary CX3CL1 may be a promising early diagnostic marker for aMCI and AD. These results could offer a simpler and more accessible approach to aMCI and AD diagnosis within the relevant research domain. Further longitudinal studies are essential to elucidate the temporal changes in CX3CL1 levels across different stages of cognitive decline and to explore its predictive value in identifying individuals at risk of developing AD.

## Data Availability

The original contributions presented in the study are included in the article/supplementary material, further inquiries can be directed to the corresponding author.
