# Supplementary material for: Diagnostic potential of urinary CX3CL1 for amnestic mild cognitive impairment and Alzheimer’s disease
Source: Front Aging Neurosci. 2025 Jan 23;17:1501762. doi: 10.3389/fnagi.2025.1501762 (PMC11798980; doi:10.3389/fnagi.2025.1501762)
Supplement: Supplementary file 1 [file Data_Sheet_1.docx]

**Diagnostic potential of urinary CX3CL1 for amnestic mild cognitive impairment and Alzheimer’s disease**

Yali Xu 1,2*, Jie Zhang 1,2, Ying-Ying Shen 3, Wei-Wei Li 3, Bin Li 4, Hai-Ping Cheng 4 and Gui-Hua Zeng 3

1 Department of Geriatrics, Chongqing General Hospital, Chongqing University, Chongqing, China, 2 Chongqing Clinical Research Centre for Geriatric Diseases, Chongqing, China, 3 Department of Neurology and Center for Clinical Neuroscience, Daping Hospital, Army Medical University, Chongqing, China, 4 Department of Health Management, Chongqing General Hospital, Chongqing University, Chongqing, China

*CORRESPONDENCE ：Yali Xu Email: xuyaliby@126.com

**Supplemental information**

**Supplemental table 1. Age and sex of the cognitively normal subjects**

|  | Sex | | | Average age (years) | | |
| --- | --- | --- | --- | --- | --- | --- |
| Age range  N=516 | Female (n, %)  n=251 (48.64) | Male (n, %)  n=265 (51.36) | P value | Female  n=251 | Male  n=265 | P value |
| 18-29 years, n=77 | 34 (44.16) | 43 (55.84) | 0.703* | 23.88 ± 0.5770 | 23.72±0.5148 | 0.8315 |
| 30-39 years, n=85 | 37 (43.53) | 48 (56.47) |  | 33.97 ±0.4642 | 34.13 ± 0.4196 | 0.8345 |
| 40-49 years, n=79 | 42 (53.16) | 37 (46.84) |  | 45.38 ± 0.4173 | 44.92 ± 0.4542 | 0.4149 |
| 50-59 years, n=116 | 59 (50.86) | 57 (49.14) |  | 54.58 ± 0.3723 | 54.88± 0.3862 | 0.5733 |
| 60-69 years, n=105 | 50 (47.62) | 55 (52.38) |  | 64.10 ±0.3761 | 63.98 ± 0.4140 | 0.7932 |
| 70-75 years, n=54 | 29 (53.70) | 25 (46.30) |  | 72.10 ±0.3113 | 71.96 ±0.3484 | 0.7293 |

* denotes comparison of the ratio of female or male among different age groups using Chi-square test.

**Supplemental table 2. Discriminative values of urinary CX3CL1 using ROC curve analysis**

| ROC analysis | AD *vs.* CN | AD *vs.* aMCI | aMCI *vs.* CN | aMCI *vs.* AD & CN | AD vs. aMCI & CN |
| --- | --- | --- | --- | --- | --- |
| AUC | 0.6174 | 0.7412 | 0.6452 | 0.6954 | 0.6683 |
| P value | 0.005 | <0.001 | 0.002 | <0.001 | <0.001 |
| 95% CI | 0.5384-0.6964 | 0.6674-0.815 | 0.5599-0.7304 | 0.6289-0.7618 | 0.5985-0.7381 |
| Cutoff, pg/mg | 1.727 | 1.311 | 1.256 | 1.296 | 1.516 |
| Sensitivity, % | 69.89 | 84.62 | 47.31 | 84.62 | 73.42 |
| Specificity, % | 54.90 | 63.73 | 81.54 | 54.36 | 59.80 |

**
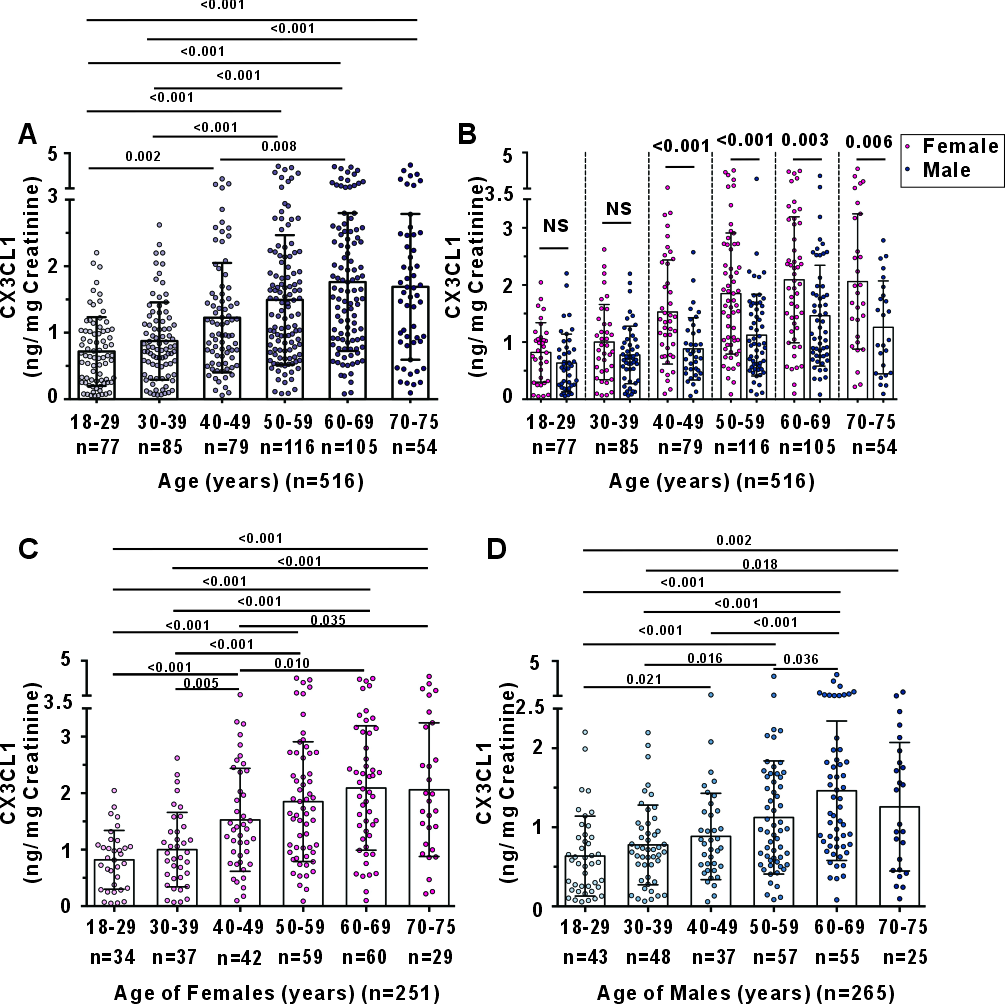
**

**Supplemental Figure 1.** Urinary CX3CL1 levels in different age groups of cognitively normal subjects. (A) Comparison of urinary CX3CL1 levels among different age groups of cognitively normal subjects. (B) Comparison of urinary CX3CL1 levels between females and males among different age groups of cognitively normal subjects. (C) Comparison of urinary CX3CL1 levels in females among different age groups of cognitively normal subjects. (C) Comparison of urinary CX3CL1 levels in males among different age groups of cognitively normal subjects. NS denotes non-statistically significant.


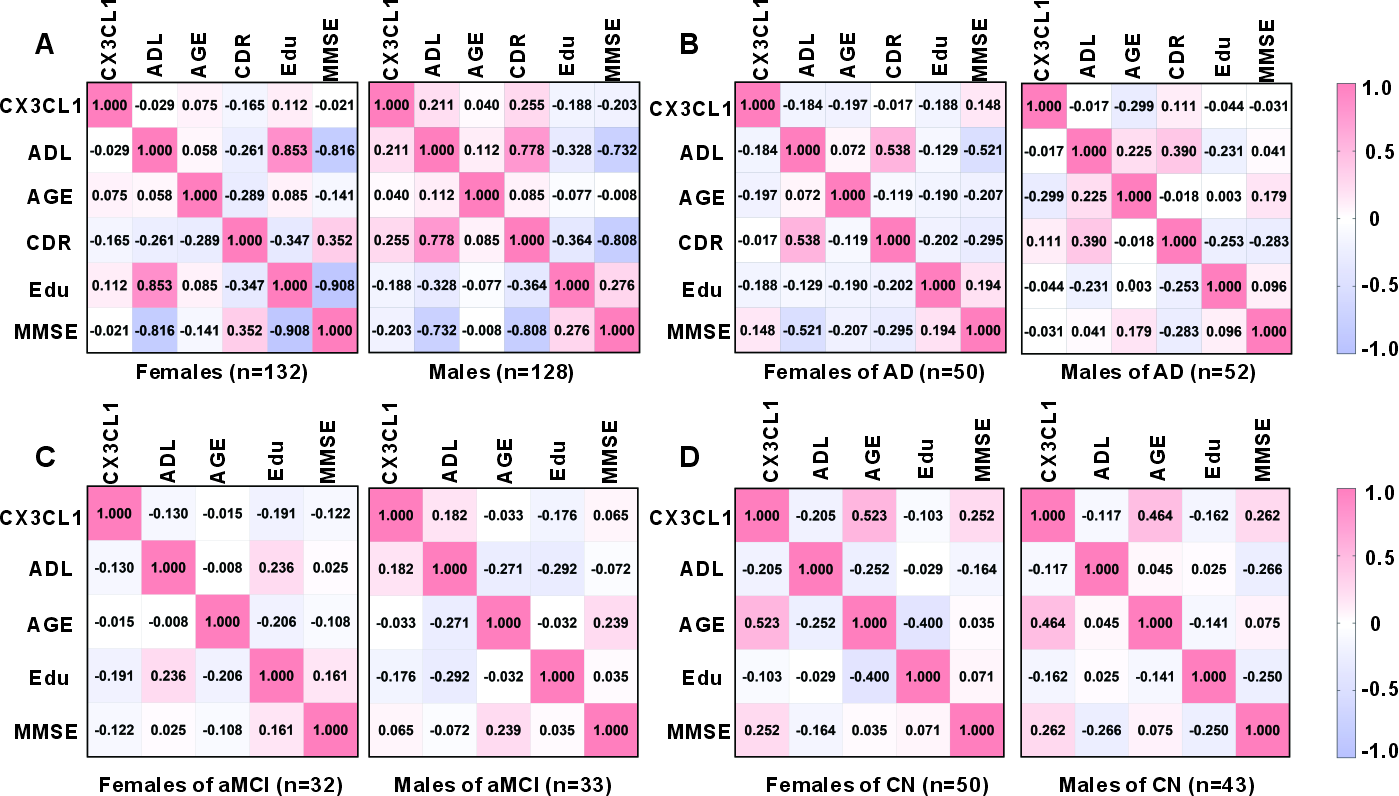


**Supplemental Figure 2.** Correlation matrix showing Spearman's correlations between urinary CX3CL1 levels, ADL scores, Age, CDR scores, Education and MMSE scores in the cohort including the AD, aMCI and CN groups.

(A) Correlation between urinary CX3CL1 levels, ADL scores, Age, CDR scores, Education and MMSE scores in females (Females) and males (Males) of the cohort including the AD, aMCI and NC groups.

(B) Correlation between urinary CX3CL1 levels ADL scores, Age, CDR scores, Education and MMSE scores in females (Females of AD) and males (Males of AD) of AD group.

(C) Correlation between urinary CX3CL1 levels ADL scores, Age, CDR scores, Education and MMSE scores in females (Females of aMCI) and males (Males of aMCI) of aMCI group.

(D) Correlation between urinary CX3CL1 levels ADL scores, Age, CDR scores, Education and MMSE scores in females (Females of CN) and males (Males of CN) of CN group.
